# Early Events in Retinal Degeneration Caused by Rhodopsin Mutation or Pigment Epithelium Malfunction: Differences and Similarities

**DOI:** 10.3389/fnana.2017.00014

**Published:** 2017-03-06

**Authors:** Johnny Di Pierdomenico, Diego García-Ayuso, Isabel Pinilla, Nicolás Cuenca, Manuel Vidal-Sanz, Marta Agudo-Barriuso, María P. Villegas-Pérez

**Affiliations:** ^1^Departamento de Oftalmología, Facultad de Medicina, Universidad de Murcia and Instituto Murciano de Investigación Biosanitaria Virgen de la ArrixacaMurcia, Spain; ^2^Instituto de Investigación Sanitaria Aragón, Aragon Health Sciences Institute, Lozano Blesa University HospitalZaragoza, Spain; ^3^Departamento de Fisiología, Genética y Microbiología, Universidad de AlicanteAlicante, Spain

**Keywords:** retina, inherited retinal degeneration, Royal College of Surgeons, P23H-1, microglia, glial fibrillary acidic protein, Müller cells, rat

## Abstract

To study the course of photoreceptor cell death and macro and microglial reactivity in two rat models of retinal degeneration with different etiologies. Retinas from P23H-1 (rhodopsin mutation) and Royal College of Surgeon (RCS, pigment epithelium malfunction) rats and age-matched control animals (Sprague-Dawley and Pievald Viro Glaxo, respectively) were cross-sectioned at different postnatal ages (from P10 to P60) and rhodopsin, L/M- and S-opsin, ionized calcium-binding adapter molecule 1 (Iba1), glial fibrillary acid protein (GFAP), and proliferating cell nuclear antigen (PCNA) proteins were immunodetected. Photoreceptor nuclei rows and microglial cells in the different retinal layers were quantified. Photoreceptor degeneration starts earlier and progresses quicker in P23H-1 than in RCS rats. In both models, microglial cell activation occurs simultaneously with the initiation of photoreceptor death while GFAP over-expression starts later. As degeneration progresses, the numbers of microglial cells increase in the retina, but decreasing in the inner retina and increasing in the outer retina, more markedly in RCS rats. Interestingly, and in contrast with healthy animals, microglial cells reach the outer nuclei and outer segment layers. The higher number of microglial cells in dystrophic retinas cannot be fully accounted by intraretinal migration and PCNA immunodetection revealed microglial proliferation in both models but more importantly in RCS rats. The etiology of retinal degeneration determines the initiation and pattern of photoreceptor cell death and simultaneously there is microglial activation and migration, while the macroglial response is delayed. The actions of microglial cells in the degeneration cannot be explained only in the basis of photoreceptor death because they participate more actively in the RCS model. Thus, the retinal degeneration caused by pigment epithelium malfunction is more inflammatory and would probably respond better to interventions by inhibiting microglial cells.

## Introduction

Inherited retinal degenerative diseases are a major cause of blindness in the world. There are many forms of the disease (OMIM; http://omim.org/) that have been associated with more than 200 different genes (RetNet; https://sph.uth.edu/retnet/). For practical purposes they are commonly divided into monogenic (mendelian) or multifactorial (complex) diseases. Age-related macular degeneration is the most common form of multifactorial retinal degeneration (Fritsche et al., 2014), while Retinitis Pigmentosa (RP) is the commonest form of the monogenic disease (Hartong et al., 2006; Swaroop and Sieving, 2013). Monogenic or mendelian retinal degenerations are also named hereditary retinal degenerations (HRD) and represent a very heterogeneous group of diseases that are relatively common, as they affect one in 2,000–3,000 individuals (Hartong et al., 2006; Daiger et al., 2013). These diseases are usually distinguished attending to their mode of inheritance (dominant, recessive, or X-linked) and to their clinical phenotype, which are the result of the different genetic mutations that cause the disease that can primarily affect rod, cones, or the retinal pigmented epithelium (RPE), necessary for normal functioning of both rods and cones (Hartong et al., 2006; Wright et al., 2010; Daiger et al., 2013; Swaroop and Sieving, 2013). However, mutations of the same gene can cause different clinical phenotypes and similar clinical phenotypes can be the result of mutations of different genes (Hartong et al., 2006; Wright et al., 2010; Daiger et al., 2013; Swaroop and Sieving, 2013).

There are many animal models of RP, and some of them have the same genetic defects observed in RP patients (LaVail, 1981; Slijkerman et al., 2015; Veleri et al., 2015). In this study, we will use two of these models: the P23H rat and the Royal College of Surgeons (RCS) rats. The P23H rat bears an autosomal dominant mutation in the rhodopsin gene (Steinberg et al., 1996; Lewin et al., 1998; LaVail et al., 2000; Machida et al., 2000). This genetic defect is a common etiology of autosomal dominant RP (Hartong et al., 2006) and causes defective disc formation, protein misfolding, endoplasmic reticulum stress, and photoreceptor degeneration (Steinberg et al., 1996; Machida et al., 2000; Veleri et al., 2015). The RCS rat bears a mutation of a RPE specific protein: MERTK, which is a tyrosine-protein kinase MER that has been also found in humans with early onset retinal degeneration and Leber Congenital Amaurosis (Gal et al., 2000; Veleri et al., 2015; Parinot and Nandrot, 2016). In this model, photoreceptor degeneration is the result of the failure of the RPE cells to phagocytize outer segment debris (LaVail, 1981; Duncan et al., 2003; Veleri et al., 2015; Parinot and Nandrot, 2016). In the first animal model, rods are affected first while in the second, as the defect is manifested in the RPE, both rods and cones are affected.

Macroglial retinal cells: astrocytes and Müller cells, readily respond to intravitreral injections (Di Pierdomenico et al., 2016), injury or disease (Ramírez et al., 2010; Gallego et al., 2012; Rojas et al., 2014; de Hoz et al., 2016). Macroglial hypertrophy has been related to retinal stress or injury, and thus, gliosis has been used to assess the homeostatic state of the retina (Cuenca et al., 2014).

Microglial cells are the resident immune cells of the central nervous system. In the healthy retina, microglial cells are located predominantly in the ganglion cell layer and in both plexiform layers (Terubayashi et al., 1984; Boya et al., 1987; Ashwell et al., 1989; Thanos, 1992; Salvador-Silva et al., 2000; Sobrado-Calvo et al., 2007). In homeostasis, microglial cells constantly survey the environment and play critical functions intervening in axonal growth, synaptic remodeling, and neuronal survival via phagocytosis of cellular debris and secretion of several cell signaling factors (Hume et al., 1983; Thanos, 1992; Bodeutsch and Thanos, 2000; Langmann, 2007; Sobrado-Calvo et al., 2007; Jonas et al., 2012; Cuenca et al., 2014; Vecino et al., 2016). Surveying microglia shows a ramified morphology characterized by a small, round soma, and various branching processes. After a negative stimulus or an insult, microglial cells change to an activated state, shortening and widening their processes and releasing a new range of inflammatory mediators (Thanos, 1992; Thanos and Richter, 1993; Salvador-Silva et al., 2000; García-Valenzuela et al., 2005; Sobrado-Calvo et al., 2007; Beynon and Walker, 2012; Jonas et al., 2012; Karlstetter et al., 2015). In animal models of inherited photoreceptor degeneration, photoreceptor death triggers the activation of microglial cells and their migration to the outer retina to phagocytose dying photoreceptors and eliminate cellular debris (Thanos, 1992; Thanos and Richter, 1993; Roque et al., 1996; Noailles et al., 2014; Zhao et al., 2015). In performing these functions, retinal microglial cells may thus influence photoreceptor survival or death (Langmann, 2007; Yoshida et al., 2013; Karlstetter et al., 2015; Li et al., 2015; Madeira et al., 2015; Zhao et al., 2015). Furthermore, recent reports have documented a correlation of pro-inflammatory cytokine levels with photoreceptor degeneration in RCS (Liu et al., 2013) and P23H-3 rats (Noailles et al., 2016) as well as inflammasome activation in P23H rats (Viringipurampeer et al., 2016), Also, there are some reports of photoreceptor rescue in inherited retinal degeneration by inhibition of microglial cells (Adamus et al., 2012; Iezzi et al., 2012; Peng et al., 2014). All these data illustrate the importance of inflammation and microglial cells in the animal models used in this study.

To the best of our knowledge there are no any detailed studies comparing the loss of photoreceptors in parallel with the activation of glial cells in the early stages of inherited retinal degenerations caused by different mechanisms and/or genetic defects. Thus, in the present study we compare and study the course of photoreceptor death and the macro and microglial cell changes in two different animal models of inherited retinal degeneration: the P23H-1 and the RCS rats, and we document a differential involvement of the microglial cells.

## Materials and methods

### Animals

We have used homozygous albino female P23H line 1 rats and homozygous pigmented Royal College of Surgeons (RCS) rats of different postnatal (P) ages: P10, 15, 21, 28, and 45 days for P23H-1 rats and P10, 21, 33, 45, and 60 for RCS rats (*n* = 6 for each strain and age). P10 is the younger age analyzed in both models because at this age retinal degeneration has not yet started. The other age-periods analyzed were chosen in order to include the ages in which it takes place the most severe part of the retinal degeneration in both models. We have also used age-matched albino female Sprague-Dawley (SD) and pigmented Piebald Virol Glaxo (PVG) rats (*n* = 6 for each strain and age analyzed) as controls for P23H-1 rats and RCS rats, respectively. Transgenic homozygous P23H-1 animals were obtained from M. LaVail (University of California at San Francisco School of Medicine; http://grantome.com/grant/NIH/R01-EY006842-25; Steinberg et al., 1996), and bred at the University of Murcia; RCS, SD, and PVG rats were obtained from the breeding colony of the University of Murcia, Spain. Rats were housed in temperature- and light-controlled rooms with a 12-h light/dark cycle (light from 8 a.m.–8 p.m.) and had food and water *ad libitum*. Animal manipulations were carried out following the Spanish and European Union regulations for the use of animals in research (Council Directive 86/609/EEC) and the ARVO statement for the use of animals in ophthalmic and vision research.

### Tissue processing

Rats were sacrificed with a lethal dose of sodium pentobarbital (Dolethal Vetoquinol, S.A., Lure, France) and perfused transcardially through the ascending aorta first with saline and then with 4% paraformaldehyde in 0.1 M phosphate buffer (pH 7.4).

### Cross-sections

The eyes were enucleated and the conjunctiva of the superior pole was marked with a 6/0 silk suture. The cornea and the lens were removed and the resulting eyecups were postfixed in 4% paraformaldehyde for 1 h. The eyecups were cryoprotected by immersion first in phosphate buffered saline (PBS) containing 15% of sucrose (Sigma, Alcobendas, Madrid, Spain) for 1 day and later in PBS containing 30% sucrose for another day. The eyecups were included in Tissue-Tek® (OCT; Sakura Finetek, Torrance, CA, USA) maintaining their orientation and rapidly frozen by immersion in isopentane cooled at −70°C. Sagital 15 microns thick cryostat cross sections of the eyecups were obtained on a cryostat (Leica Jung CM3000), and placed ordered in rows in VWR® SuperFrost® Plus slides (VWR International bvba, Galdenaaksebaan, Leuven, Belgium), where they were processed for TdT-mediated dUTP nick-end labeling (TUNEL) to label apoptotic nuclei (García-Ayuso et al., 2011; Montalbán-Soler et al., 2012) or for immunohistofluorescence.

### Immunohistofluorescence

Cross-sections were washed three times in PBS containing 0.1% Triton X-100 (Tx; Sigma-Aldrich, Alcobendas Madrid, Spain) to eliminate the embedding medium and incubated overnight at 4°C with a mixture of the primary antibodies (see next paragraph) diluted in blocking buffer (PBS containing 2% Tx and 2% normal donkey or goat serum, depending on the origin of the secondary antibody used). After washing with PBS-0.1%Tx the sections were incubated for 1 h at room temperature with a mixture of the secondary antibodies (see next paragraph) diluted in PBS-2%Tx. Sections were rinsed again with PBS-0.1%Tx and then mounted with a mounting media containing DAPI (4′,6-diamidino-2-phenylindole; Vectashield Mounting Medium con DAPI, Vector Atom, Alicante, España) to counterstain all retinal nuclei. For antigen retrieval, the sections processed for PCNA immunohistofluorescence were washed with PBS and kept overnight at 40°C (in top of a water bath) with target retrieval solution (S1699, Dako, Santa Clara, California, USA) 1:10 in distilled water.

### Antibodies

#### Primary antibodies

Microglial cells were detected by using rabbit anti-Iba1 antibody (1:1000; 019-19741: Wako Chemicals, USA). Astrocytes and Müller cells were detected by using goat anti-GFAP antibody (1:250; C-19: sc-6170; Santa Cruz Biotechnology, Heidelberg, Germany). To detect the outer segment of rods and cones: rabbit anti-red/green opsin (1:1200; AB5405; Chemicon-Millipore Iberica, Madrid, Spain), goat anti-blue opsin (1:1000; N-20; OPN1SW; Santa Cruz Biotechnology, Heidelberg, Germany) and mouse anti-rhodopsin (1:1200, 1D4; Sigma-Aldrich, Madrid, Spain) antibodies were used. Cellular proliferation and blood vessels were detected with mouse anti-PCNA (Proliferating Cell Nuclear Antigen; 1:50, PC10: sc-56, Santa Cruz Biotechnology, Heidelberg, Germany) and Isolectin GS-IB4 from Griffonia Simplicifolia conjugated to Alexa-fluor-568 (1:200; Invitrogen, Molecular Probes, Oregon, USA), respectively.

#### Secondary antibodies

Donkey anti-goat Alexa Fluor® 594 conjugate, donkey anti-mouse Alexa Fluor® 594 conjugate, and donkey anti-rabbit Alexa Fluor® 488 conjugate (all from: Molecular Probes, Invitrogen Inc., Madrid, Spain) and diluted at 1:500.

In each animal, alternate sections containing the optic disk were incubated either with anti-red/green and blue opsin, with anti-rhodopsin, with anti-Iba-1, and anti-GFAP antibodies or with anti-PCNA and lectin GS-IB4 or Iba-1 or GFAP.

### Image analysis

In each animal, three sagittal cross sections containing the optic disk (from the nasal, central, and temporal part of the optic disk) and stained with different antibodies were selected attending to the quality of the section, examined and photographed under a fluorescence microscope (Axioscop 2 Plus; Zeiss Mikroskopie, Jena, Germany) equipped with various filters and a digital high-resolution camera (ProgRes C10; Jenoptik, Jena, Germany). Eight photographs (4 from the dorsal retina and 4 from the ventral retina X20, measuring 530 × 390 μm) were taken with the blue (in order to observe the DAPI-stained nuclei), green and/or red filters in every section at different distances between the optic nerve and the retinal periphery. Because during the time of the study the eye of the animals grows, and in order to compare corresponding regions at the different ages studied, these distances were not absolute measurements, but represented similar retinal regions. To that end, the distance between the optic disc and the retinal periphery (both in the dorsal and ventral retina) was measured and the four photomicrographs were taken at distances representing 25, 50, 75, and 95% of the length between the optic disk and the retinal periphery (both in the dorsal and ventral retina). Images were further processed using Adobe® Photoshop® CS 6 (Adobe Systems, Inc., San Jose, CA, USA) when needed.

### Qualitative analysis of rods and cones in cross sections

The density, morphology, and length of rod and cone outer segments was analyzed qualitatively in the fluorescence photomicrographs taken with different filters in the sections stained with anti-opsin or anti-rhodopsin antibodies.

### Quantification of the thickness of the outer nuclear layer

Outer nuclear layer thickness was manually analyzed in three regions (central, right, and left) of each photomicrograph. The number of nuclei rows of the outer nuclear layer (ONL) were counted and averaged, thus obtaining a mean number or nuclei rows per picture, per retinal region analyzed and per animal (García-Ayuso et al., 2010, 2011; Montalbán-Soler et al., 2012).

### Quantification of microglial cells

To quantify the numbers of microglial cells in the different retinal layers, the two photomicrographs taken at each chosen retinal region, one with the blue filter to observe the DAPI-labeled nuclei, and one with the green filter to observe the Alexa Fluor® 594-labeled microglial cells were merged with the Adobe® Photoshop® program. The numbers of microglial cells were subsequently counted in each photomicrograph in the following retinal layers: nerve fiber layer + ganglion cell layer (GCL), inner plexiform layer (IPL), inner nuclear layer (INL), outer plexiform layer (OPL), ONL and layer of the photoreceptor outer segments (OS). As before, these counts were pooled to obtain a mean number of microglial cells per layer, per retinal region and per animal (six animals per age were analyzed).

### Statistics

The numbers of nuclei rows in the ONL and the numbers of microglial cells were compared between retinal regions and animals. Statistical analysis was carried out using SigmaStat® 3.11 for Windows (Systat Software, Inc., Tichmond, CA, USA). The one or 2-way ANOVA test was used to compare the numbers of microglial cells between the same animals at different ages and to compare between P23H-1 and RCS and between SD and PVG animals, respectively. The *t*-test was used to compare two different groups. Differences were considered significant when *p* ≤ 0.05.

## Results

### Control albino and pigmented animals

**Figure 1:**
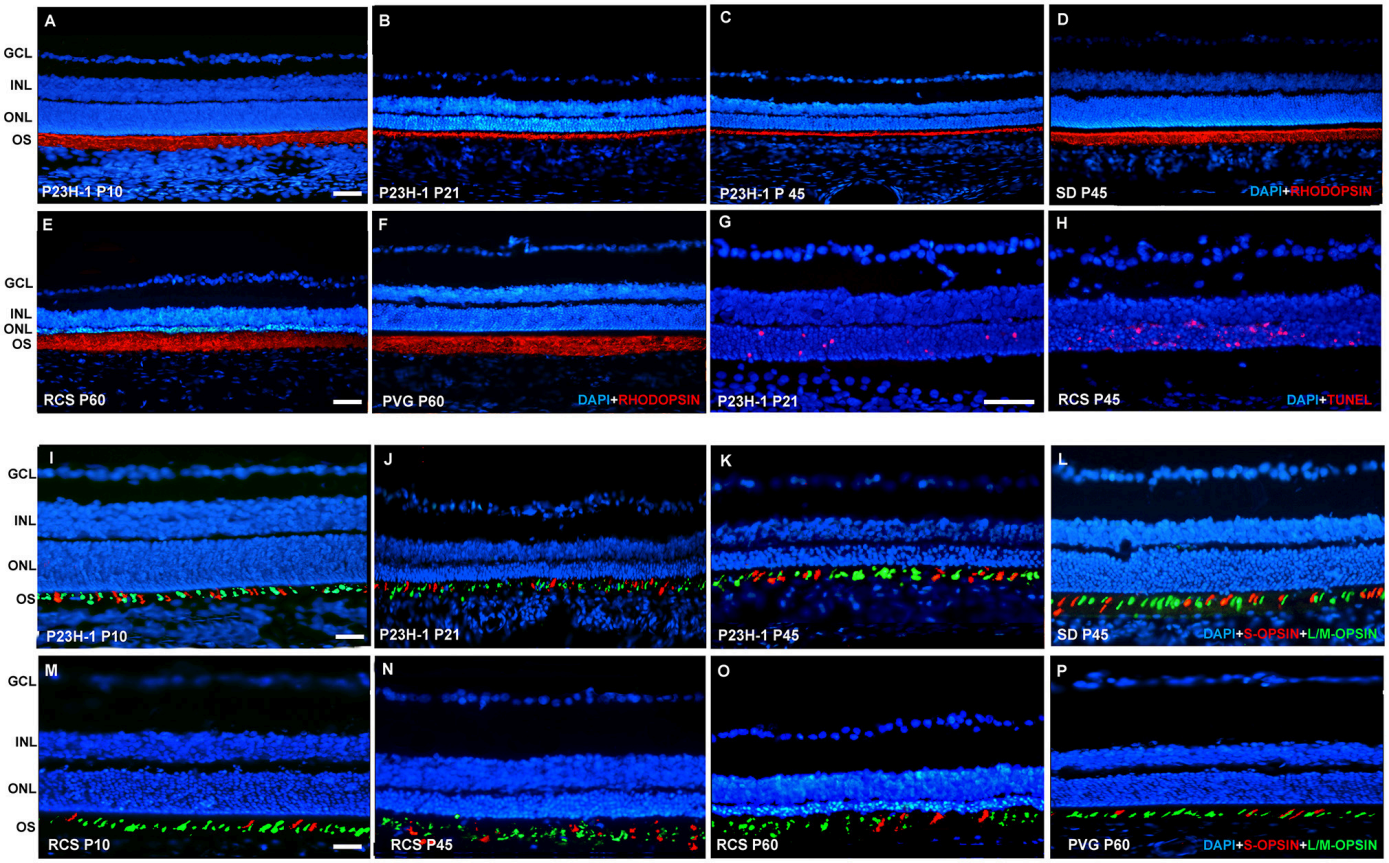
**Rhodopsin and opsin expression in control ***SD*** and PVG rats and P23H-1 and RCS rats**. Photomicrographs of representative retinal cross sections taken from the mid-dorsal retina of control *SD* **(D,L)** and PVG **(F,P)** and P23H-1 **(A–C,I–K)** and RCS **(E,M–O)** rats showing rhodopsin immunoreactivity (red) and DAPI-counterstaining (blue; **A–F**), S- (red) and L/M- (green) opsin immunoreactivity and DAPI-counterstaining (blue; **I–P**) or TUNEL labeling of photoreceptors (red) and DAPI-counterstaining (blue; **G,H**). In P23H-1 rats the expression of rhodopsin is already affected at P10 (**A**, compare to **D**), and decreases further until P45, the latest time analyzed **(B,C)**. At P10 the cone outer segments look normal (**I**, compare to **L**) but show degeneration signs at P21 and P45 **(J,K)**. In RCS rats rhodopsin expression did not change at any of the time points studied (**E**, compare to **F**). The outer segments of cones look normal at P10 (**M**, compare to **P**) but at P45 and P60 **(N,O)** they are degenerated. TUNEL staining reveals abundant photoreceptor apoptosis during retinal degeneration in P23H-1 and RCS rats **(G,H)**. Scale bar = 100 μm.

**Figure 2:**
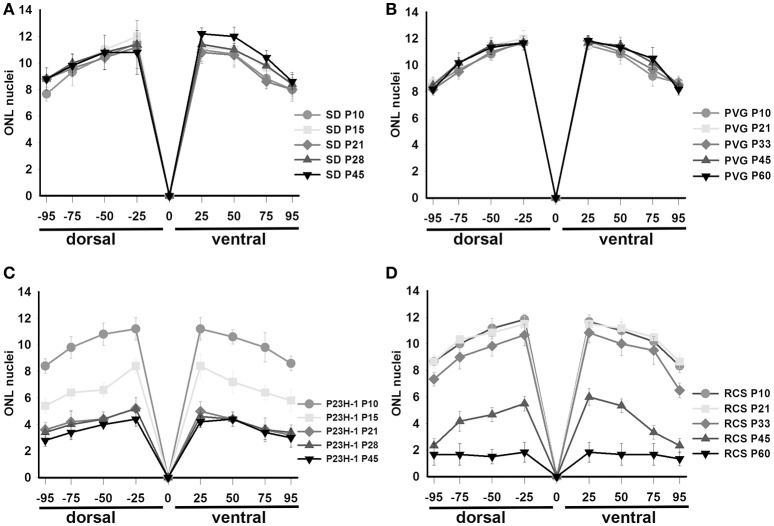
**Thickness of the ONL in control and dystrophic animals**. Graphs showing the mean (±*SD*) numbers of nuclei rows in the ONL of control *SD* **(A)** and PVG **(B)** rats and dystrophic P23H-1 **(C)** and RCS **(D)** rats of different ages (see insert). The numbers of rows were counted manually in pictures taken at four different distances from the optic disk (see Section Materials and Methods).

Rhodopsin and opsins are expressed in the outer segments (OS) of rods and S- or L/M-cones, respectively. In control animals of both strains, the OS are elongated and qualitatively their length, density, and morphology does not change with age (Figure 1 shows the oldest retinas, younger ones not shown). The thickness of the outer nuclear layer (ONL) varies between 8 and 12 nuclei depending on the retinal region considered, decreasing from the optic nerve to the periphery (Figures 2A,B; Table 1).

**Table 1:** **Average of number of nuclei in ONL with age (mean number ± standard deviation)**.

	**DORSAL**				**O.N**.	**VENTRAL**			
	**95%**	**75%**	**50%**	**25%**	**0%**	**25%**	**50%**	**75%**	**95%**
SD P10	7.6 ± 0.4	9.3 ± 0.9	10.5 ± 0.5	11.5 ± 0.5	0	11 ± 0.8	10.8 ± 0.8	8.8 ± 0.7	8.1 ± 0.8
SD P15	8.8 ± 0.7	9.8 ± 0.4	11 ± 0.8	12 ± 0.6	0	11.2 ± 0.7	11 ± 0.8	9.7 ± 0.8	8.5 ± 0.8
SD P21	8.9 ± 0.7	9.7 ± 0.4	10.4 ± 0.8	11.4 ± 0.7	0	10.8 ± 0.7	10.5 ± 0.8	8.6 ± 0.5	8 ± 0.6
SD P28	9 ± 0.8	10 ± 0.6	10.8 ± 1.3	11.5 ± 1.5	0	11.3 ± 0.7	11 ± 0.7	9.8 ± 0.7	8.5 ± 0.8
SD P45	8.5 ± 1	9.6 ± 0.8	10.6 ± 0.7	10.8 ± 1.4	0	11.6 ± 1.2	11.8 ± 0.7	10.5 ± 0.5	8.8 ± 0.7
PVG P10	8.5 ± 0.5	9.7 ± 0.5	10.8 ± 0.4	11.8 ± 0.7	0	11.5 ± 0.5	10.8 ± 0.7	9.1 ± 0.7	8.7 ± 0.5
PVG P21	8.7 ± 0.4	10.2 ± 0.7	11.5 ± 0.5	12 ± 0.6	0	11.5 ± 0.5	11.3 ± 0.8	10.3 ± 0.5	8.5 ± 0.5
PVG P33	8.16 ± 0.4	9.5 ± 0.5	11 ± 0.6	11.7 ± 0.5	0	11.8 ± 0.4	11 ± 0.6	9.6 ± 0.5	8.5 ± 0.4
PVG P45	8.5 ± 0.5	10.2 ± 0.7	11.5 ± 0.5	11.7 ± 0.5	0	11.7 ± 0.5	11.5 ± 0.5	10.1 ± 0.4	8.5 ± 0.5
PVG P60	8.2 ± 0.4	10.2 ± 0.4	11.3 ± 0.5	11.7 ± 0.5	0	11.8 ± 0.4	11.3 ± 0.5	10.5 ± 0.8	8.2 ± 0.4
P23H-1 P10	8.5 ± 0.5	9.8 ± 0.7	10.6 ± 0.7	11.1 ± 0.83	0	11.2 ± 0.8	10.5 ± 0.5	9.8 ± 0.9	8.5 ± 0.5
P23H-1 P15	5.5 ± 0.5^*^	6.3 ± 0.5^*^	6.5 ± 0.5^*^	8.3 ± 0.5^*^	0	8.2 ± 0.9^*^	7.3 ± 0.8^*^	6.3 ± 0.5^*^	5.8 ± 0.7^*^
P23H-1 P21	3.5 ± 0.5^*^	4.3 ± 0.8^*^	4.3 ± 0.5^*^	5.2 ± 0.7^*^	0	5 ± 0.6^*^	4.5 ± 0.5^*^	3.6 ± 0.8^*^	3.2 ± 0.4^*^
P23H-1 P28	3.3 ± 0.5	4 ± 0.9	4.3 ± 0.5	5.2 ± 0.7	0	4.6 ± 0.5	4.5 ± 0.5	3.6 ± 0.7	3.3 ± 0.5
P23H-1 P45	2.8 ± 0.4	3.3 ± 0.5	4.2 ± 0.1	4.3 ± 0.5	0	4.3 ± 0.5	4.3 ± 0.5	3.5 ± 0.5	2.8 ± 0.7
RCS P10	8.7 ± 0.5	10 ± 0.6	11.16 ± 0.75	11.8 ± 0.4	0	11.7 ± 0.5	11 ± 0.6	10.2 ± 0.4	8.3 ± 0.5
RCS P21	8.7 ± 0.5	10.3 ± 0.5	10.83 ± 0.75	11.5 ± 0.54	0	11.5 ± 0.5	11.2 ± 0.7	10.5 ± 0.5	8.7 ± 0.5
RCS P33	7.3 ± 0.5^*^	9 ± 0.8^*^	9.83 ± 0.75^*^	10.7 ± 0.7^*^	0	10.8 ± 0.7^*^	10 ± 0.8^*^	9.5 ± 1^*^	6.5 ± 0.5^*^
RCS P45	2.3 ± 0.5^*^	4.1 ± 0.7^*^	4.66 ± 0.51^*^	5.5 ± 0.5^*^	0	6 ± 0.6^*^	5.3 ± 0.5^*^	3.3 ± 0.5^*^	2.3 ± 0.5^*^
RCS P60	1.7 ± 0.8	1.7 ± 0.7^*^	1.5 ± 0.54^*^	1.9 ± 0.7^*^	0	1.9 ± 0.7^*^	1.6 ± 0.7^*^	1.7 ± 0.8^*^	1.3 ± 0.5^*^

* *Statistical difference with the previous time point studied (examined in the same area). p ≤ 0.05(t-test)*. *n = 6 for all groups at all ages analyzed*.

**Figure 3:**
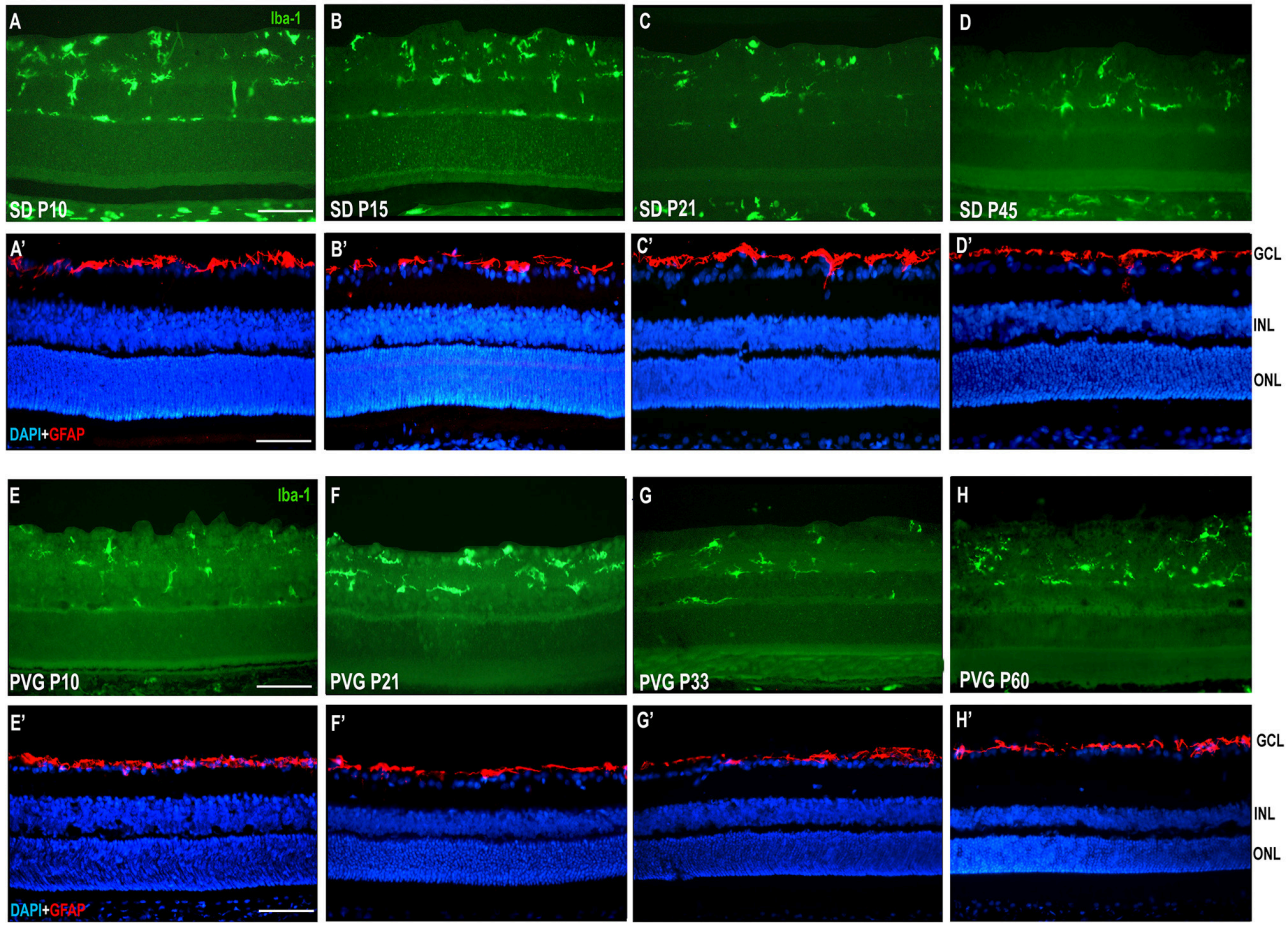
**Microglial cells and GFAP expression in control SD and PVG rats**. Photomicrographs from the mid-dorsal retina of representative cross sections immunoreacted with antibodies against Iba1 (first and third row) or GFAP and DAPI counterstaining (second and fourth row) showing microglial cells (Iba1^+^, green, **A–D,E–H**), astrocytes (GFAP^+^, red, **A'–D',E'–H'**) and DAPI-labeled nuclei (blue, **A'–D',E'–H'**). Microglial cell appear in different retinal layers while GFAP expression is limited to astrocytes situated in the innermost retinal layers. The thickness of the ONL in varies between 8 and 12 rows of nuclei and is similar at all ages. Scale bar = 100 μm.

**Figure 4:**
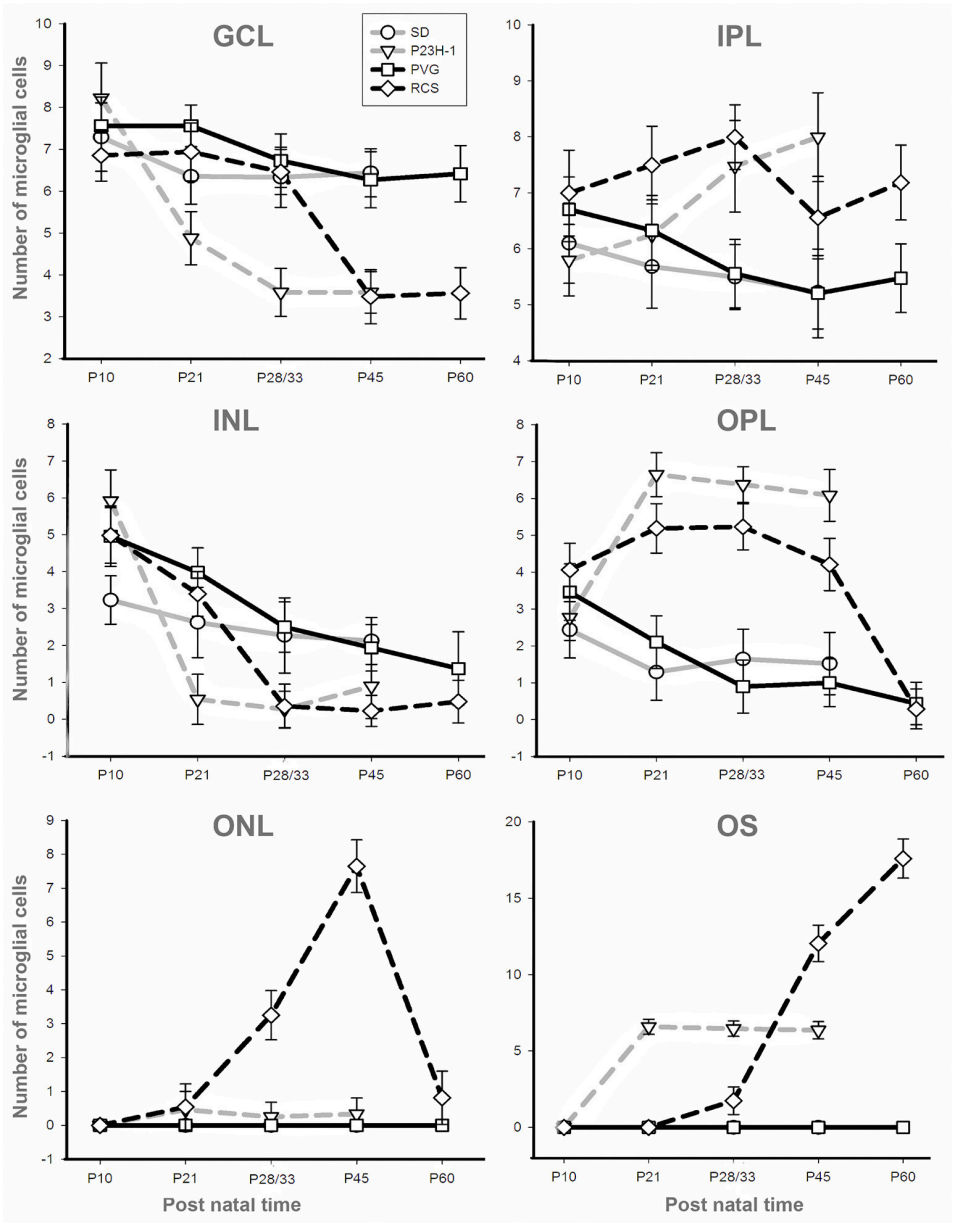
**Numbers of microglial cells in the different retinal layers**. Mean numbers (±*SD*) of microglial cells counted in each retinal layer at the different ages analyzed in *SD* rats (gray solid), P23H-1 rats (gray interrupted), PVG rats (black solid), and RCS rats (black interrupted; see also insert). Control animals show sustained numbers of microglial cells in the inner retinal layers, decreasing numbers in the INL and OPL and absence of microglial cells in the ONL and OS layer. Dystrophic animals show decreasing numbers of microglial cells in the GCL and INL and increasing numbers in the OPL and OS layer. Dystrophic animals show a striking difference in the ONL: the number of microglial cells increase in RCS rats but are almost absent in P23H-1 rats.

Surveying microglial cells are found in all retinal layers except the ONL and OS layers (Figures 3, 4). Microglial cell number varies among layers, in the GCL and IPL we found a mean of 6–9 cells, and in the INL and OPL a mean of 0–5 (Figure 4, Table 2).

**Table 2:** **Numbers of microglial cells in the different retinal layers (mean numbers ± standard deviation)**.

**A**	***SD*** **P10**	***SD*** **P15**	***SD*** **P21**	***SD*** **P28**	***SD*** **P45**	**P23H-1 P10**	**P23H-1 P15**	**P23H-1 P21**	**P23H-1 P28**	**P23H-1 P45**
GCL	7.3 ± 0.8	6.5 ± 0.7^†^	6.25 ± 0.6	6.3 ± 0.7	6.5 ± 0.6	8.2 ± 0.8^*^	5.2 ± 0.6^*^^†^	4.7 ± 0.6^*^^†^	3.5 ± 0.6^*^^†^	3.8 ± 0.5^*^
IPL	6.1 ± 0.7	6.15 ± 0.8	5.6 ± 0.7	5.5 ± 0.6	5.13 ± 0.7	5.8 ± 0.6^#^	6.8 ± 0.6^*^^†^	6.5 ± 0.6^*^^†^	7.5 ± 0.8^*^^†^	8 ± 0.8^*^^†^
INL	3.1 ± 0.5	3.55 ± 0.7	2.25 ± 0.9^†^	2.27 ± 1	2.12 ± 0.6	6.01 ± 0.8^*^	2.2 ± 0.6^*^^†^	0.5 ± 0.7^*^^†^	0.2 ± 0.5^*^	0.9 ± 0.9^*^^†^
OPL	2.4 ± 0.6	2.3 ± 0.7	1.2 ± 0.7^†^	1.4 ± 0.8	1.4 ± 0.9	2.7 ± 0.6^*^	4.3 ± 0.9^*^	6.8 ± 0.6^*^^†^	6.7 ± 0.5^*^	6.3 ± 0.7^*^
ONL	0	0	0	0	0	0	0.4 ± 0.6^*^^†^	0.5 ± 0.5^*^	0.2 ± 0.4^*^^†^	0.3 ± 0.5^*^
OS	0	0	0	0	0	0	3.2 ± 0.8^*^^†^	6.6 ± 0.5^*^	6.5 ± 0.5^*^	6.2 ± 0.6^*^
All layers	457 ± 8	439 ± 2	383 ± 7^†^	378 ± 32	367 ± 4	509 ± 10	533 ± 10^*^^†^	616 ± 17^*^^†^	586 ± 13^*^	607 ± 7^*^
**B**	**PVG P10**	**PVG P21**	**PVG P33**	**PVG P45**	**PVG P60**	**RCS P10**	**RCS P21**	**RCS P33**	**RCS P45**	**RCS P60**
GCL	7.6 ± 0.7	7.6 ± 0.5	6.5 ± 0.6^†^	6.1 ± 0.7^†^	6.2 ± 0.7	6.7 ± 0.6^*^^#^	6.7 ± 0.7^*^^#^	6.4 ± 0.5^*^^†^^#^	3.5 ± 0.6^*^^†^	3.5 ± 0.6^*^
IPL	6.8 ± 0.6	6.2 ± 0.6	5.6 ± 0.6^†^	5.2 ± 0.8^†^	5.4 ± 0.6	7.0 ± 0.8^*^^#^	7.5 ± 0.7^*^^#^	8.2 ± 0.6^*^^#^	6.5 ± 0.7^*^^†^^#^	7.2 ± 0.7^*^^†^
INL	4.8 ± 0.8	4 ± 0.7^†^	2.7 ± 0.7^†^	1.9 ± 0.6	1.4 ± 1	4.9 ± 0.7^#^	3.6 ± 0.6^*^^†^^#^	0.3 ± 0.6^*^^†^	0.2 ± 0.4^*^^#^	0.5 ± 0.6^*^
OPL	3.3 ± 0.8	2.1 ± 0.7^†^	0.80 ± 0.7^†^	1 ± 0.6	0.5 ± 0.6^†^	4.3 ± 0.7^*^^#^	5.3 ± 0.7^*^^†^^#^	5.2 ± 0.6^*^^#^	4.2 ± 0.7^*^^†^^#^	0.3 ± 0.5^†^
ONL	0	0	0	0	0	0	0.5 ± 0.7^*^^†^	3.2 ± 0.7^*^^†^^#^	7.6 ± 0.8^*^^†^^#^	0.8 ± 0.8^*^^†^
OS	0	0	0	0	0	0	0^#^	1.7 ± 0.9^*^^†^^#^	12 ± 1.2^*^^†^^#^	17.6 ± 1.3^*^^†^
All layers	549 ± 25^#^	479 ± 12^†^^#^	376 ± 2^†^	370 ± 25	353 ± 31	570 ± 15	578 ± 7^*^	608 ± 8^*^	820 ± 21^*^^†^	718 ± 26^*^^†^^#^

* *Statistically different when compared to its respective control p < 0.05*.

† *Statistically different when compared to the previous time point studied (in the same strain and retinal layer) p < 0.05*.

# *Statistically different when compared strain SD with PVG and strainP23H-1 with RCS at the same time point in the same layer p < 0.001*.

*n = 6 for all groups at all ages analyzed*.

The mean number of microglial cells counted per retinal section (i.e., sum of microglial cells counted in all layers) varies between 350 and 600. As shown in Table 2, at P10 and P21 the pigmented strain (PVG) has a significantly higher number of microglial cells than the albino strain (SD). In addition, the numbers of microglial cells decrease with age in certain layers and at specific post-natal times: between P10–P15 and P21 in SD rats, between P10 and P21, and between P21 and P33 in PVG rats (Table 2).

**Figure 5:**
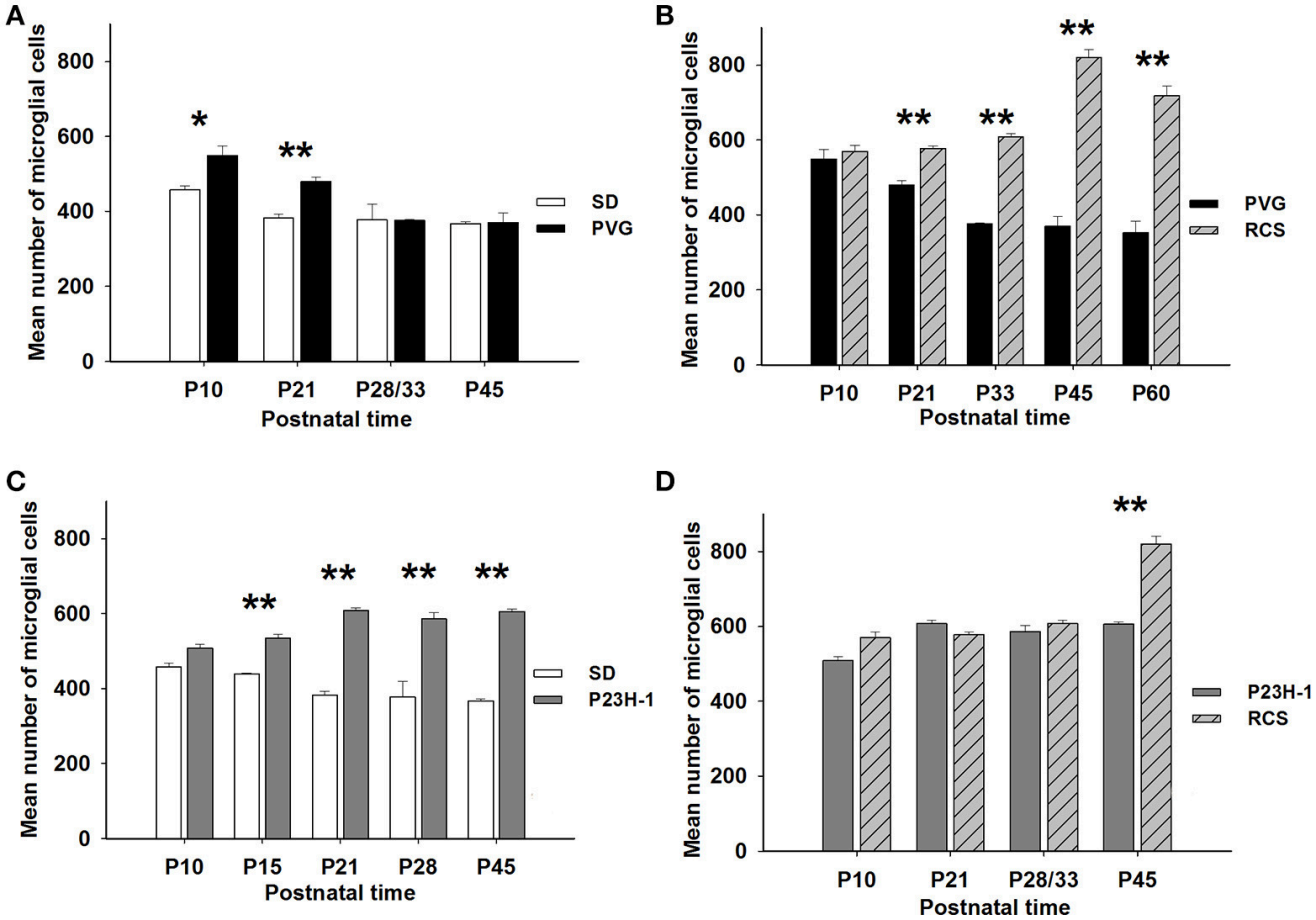
**Numbers of microglial cells in control and dystrophic animals**. Mean numbers (±*SD*) of microglial cells per group of animals at different postnatal ages and comparison between control *SD* and PVG rats **(A)**, between pigmented animals: RCS vs. PVG **(B)**, between albino animals: P23H-1 vs. *SD* **(C)**, and between dystrophic animals: P23H-1 vs. RCS **(D)**. Differences statistically significant at the same postnatal age ^*^p ≤ 0.01 and ^**^p ≤ 0.001. PVG rats have significantly more microglial cells at P10 and P21 **(A)**, RCS and P23H-1 rats have significantly more microglial cells than their control PVG and SD rats at all postnatal ages except P10 **(B,C)** and RCS rats have more microglial cells than P23H-1 rats only at P45 **(D)**.

Thereafter, from P21 in the albino (SD) or P33 in the pigmented (PVG), the total numbers of microglial cells remained stable and was similar in both strains (Figure 5, Table 2). These findings suggest that the decrease of microglial cells observed during retinal development takes place at more advanced ages in PVG than in SD rats and therefore that the adult numbers are reached later in the pigmented (PVG) animals (Figure 5, Table 2).

GFAP signal was only observed in the innermost retinal layer in control animals, and corresponds to the location of astrocytes in the ganglion cell layer (Figures 3D,H).

### P23H-1 rat

**Figure 6:**
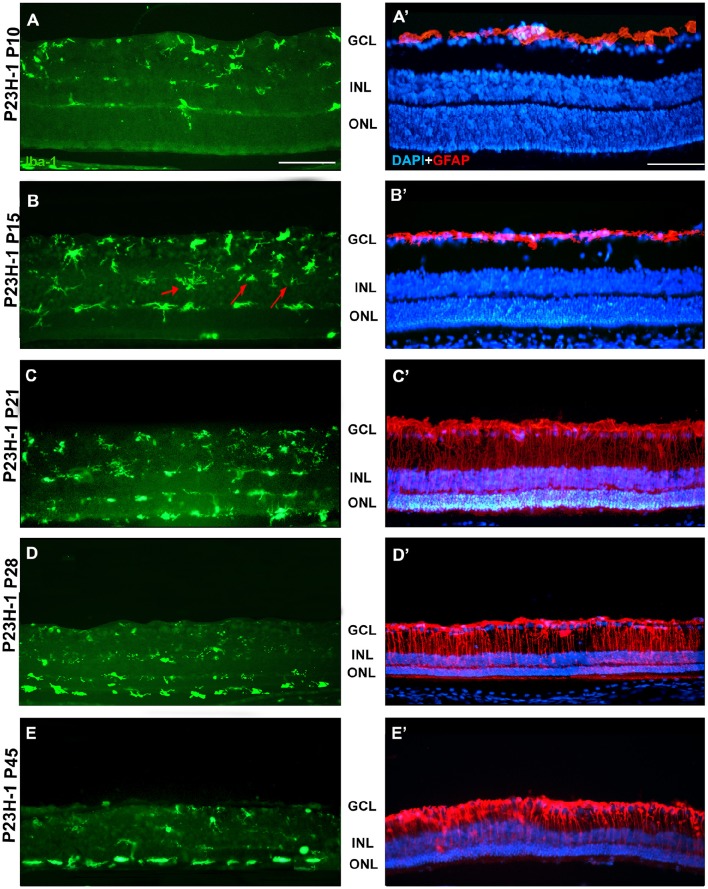
**Changes in microglial cells and GFAP expression with age in P23H-1 rats**. Photomicrographs from the mid-dorsal retina of representative cross sections of P23H-1 rats of different ages from P10 to P45, showing immunolabeled microglial cells (Iba1^+^, green, **A–E**), astrocytes and Muller cells (GFAP^+^, red), and DAPI-labeled nuclei (blue, **A'–E'**). At P15 there are some microglial cells in the INL (arrows, **B**). At P21 microglial cells migrate toward the ONL and OS layer, there is increased GFAP expression and decreased thickness of the ONL **(C,C')**, and those events continue until P45 **(D,E,D',E')**. Scale bar = 100 μm.

In the P23H-1 rat, the expression of rhodopsin and opsin was not yet affected at P10 (Figure 1) and at this age, the ONL still maintains its normal thickness (Figures 2, 6; Table 1). Although at P10 in P23H-1 retinas, the numbers of microglial cells in some layers were significantly higher than in the corresponding layers of SD retinas, there were no significant differences in their total number (Figure 5; Table 2). In agreement with the normal number of ONL nuclei rows and OS morphology, at P10 in P23H-1 rats no microglial cells were found in the ONL or OS layers.

Between P10 and P21 the outer segments of photoreceptors shortened and by P21 only a thin retinal strip remained labeled with rhodopsin in which the cone outer segments had lost their typical elongated shape and appeared fragmented (Figure 1). The numbers of nuclei row in the ONL decreased with age: from 8–12 nuclei rows at P10 to 6–9 at P15 and to around 3–6 at P21, remaining later stable up to P45, the oldest age analyzed (Figures 2, 6; Table 1). TUNEL Staining showed apoptotic nuclei in the outer nuclear layer in both models (Figures 1G,H) during the period of photoreceptor death. Coinciding with the beginning of photoreceptor degeneration (P15), there were changes in the morphology of microglial cells that showed shortened ramifications and an amoeboid morphology, suggestive of microglial activation (Figure 6B). Furthermore, by P15 the total number of microglial cells was significantly higher in P23H-1 rats than in SD rats, and this difference remained statistically significant until P45 (Figures 5C, 6; Table 2). Thus, even though photoreceptor loss did not progress from P21 in P23H-1 rats the microglial cells remained activated at least up to P45. Quantification of the numbers of microglial cells in the different retinal layers between P10 and P45 showed that their number decreased with age in the GCL and INL and increased in the IPL, OPL, ONL, and OS layers (Figures 4, 6; Table 2), suggesting that microglial cells migrated from the inner to the outer retinal layers, where cell death was occurring. Finally, GFAP immunoreactivity remained stable in astrocytes of the inner retinal layers but increased in Müller cells from P21 to P45. Thus, Müller cells were not GFAP immunoreactive in control animals but became strongly immunoreactive with age in P23H-1 rats, a clear sign of gliosis (Figure 6).

### Royal college of surgeons rat

In the RCS rat retina there were no changes in the expression pattern of rhodopsin at any of the studied ages (data non-showed; Figure 1E) i.e., the rods OS did not decrease in density and length at any time point studied, this will be discussed later as this rhodopsin signal is the debris of the rod OS that cannot be phagocytosed by the RPE. The cone OS however, showed some morphologic signs of degeneration at P45, namely shorter and distorted outer segments, and these changes persisted up to P60, the oldest age studied (Figures 1K–M).

**Figure 7:**
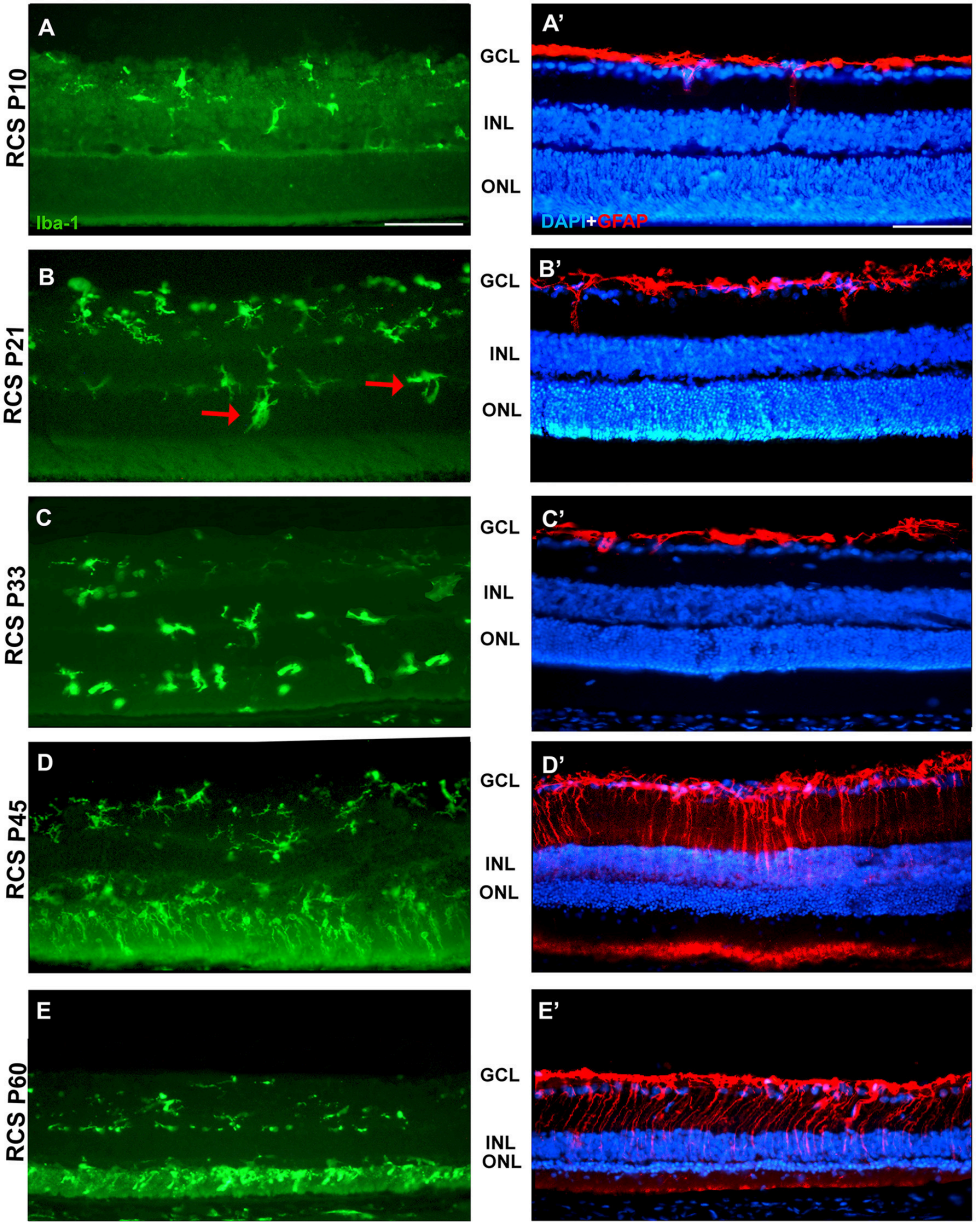
**Changes in microglial cells and GFAP expression with age in RCS rats**. Photomicrographs from the mid-dorsal retina of representative cross sections of P23H-1 rats of different ages from P10 (upper row) to P60 (lower row) showing immunolabeled microglial cells (Iba1^+^, green, **A–E**), astrocytes and Muller cells (GFAP^+^, red, **A'–E'**) and DAPI-counterstaining (blue; **A'–E'**). At P21 **(B,B')** microglial cells migrate toward the outer retinal layers (red arrows), but the expression of GFAP and the thickness of the ONL remain unchanged. At P45 there are more microglial cells in the retina and they enter the OS layer, there is overexpression of GFAP and a decrease in thickness of the ONL **(D,D')**. At P45 the thickness of the ONL has decreased further, there is more microglia in the OS layer and more GFAP expression in Müller cells **(E,E')**. Scale bar = 100 μm.

Photoreceptor degeneration was slower in RCS than in P23H rats: until P21 (Figure 7B) the number of nuclei rows in the ONL was comparable to that of control animals (8–12 rows. Figures 2, 7; Table 1. Nuclei loss in the ONL started at P33 (6–11 nuclei rows), although it was between P33 and P60 when loss was maximal, remaining only 1–2 nuclei rows at P60 (Figures 2, 7; Table 1).

In accordance to the course of photoreceptor loss, the total number of microglial cells remained constant up to P33, but their numbers increased at P45 and P60. However, at P21 and P33 some microglial cells had changed to an amoeboid morphology (Figures 7B,C). Moreover, from P21 to P60, migration of microglia was observed from the inner to the outer retinal layers (Figures 7B–E). When we compared the total number of microglial cells in RCS rats with their respective controls, PVG rats, we found that there were significantly higher numbers in RCS rats from P21 to P60 (Figure 5B; Table 2). The total numbers of microglial cells reached a maximum at P45 and between P45 and P60 there was a significant decrease, suggesting that between these two time points microglial activation was declining (Figure 5B, Table 2).

As observed in the P23H retina, the numbers of microglial cells in the outer retinal layers (OPL, ONL, and OS) increased significantly during the progression of the disease, reaching in the RCS rat their maximum: in the OPL at P33, in the ONL at P45 and in the OS layer at the oldest age studied: P60 (Figures 4, 6, 7; Table 2). At the same time, the numbers of microglial cells decreased in the inner retinal layers indicating, as in P23H-1 rats, migration of microglial cells from the inner to the outer retinal layers (Figures 4, 7). Microglial cells migrate to the OS layer to phagocytose the outer segments that cannot be phagocytosed by the RPE.

In RCS rats, GFAP expression in astrocytes of the inner retinal layers did not change with time. However, and as observed in P23H-1 retinas, GFAP expression increased importantly in Müller cells that became strongly immunoreactive with age, although this increase was found later in RCS rats, at P45 (Figure 7). Therefore, gliosis in this strain occurs later than in the P23H-1 rat (Figures 6, 7), maybe reflecting a less severe disturbance of retinal homeostasis or perhaps because the loss of photoreceptors starts later.

### Microglial cell increase is due to cellular division

**Figure 8:**
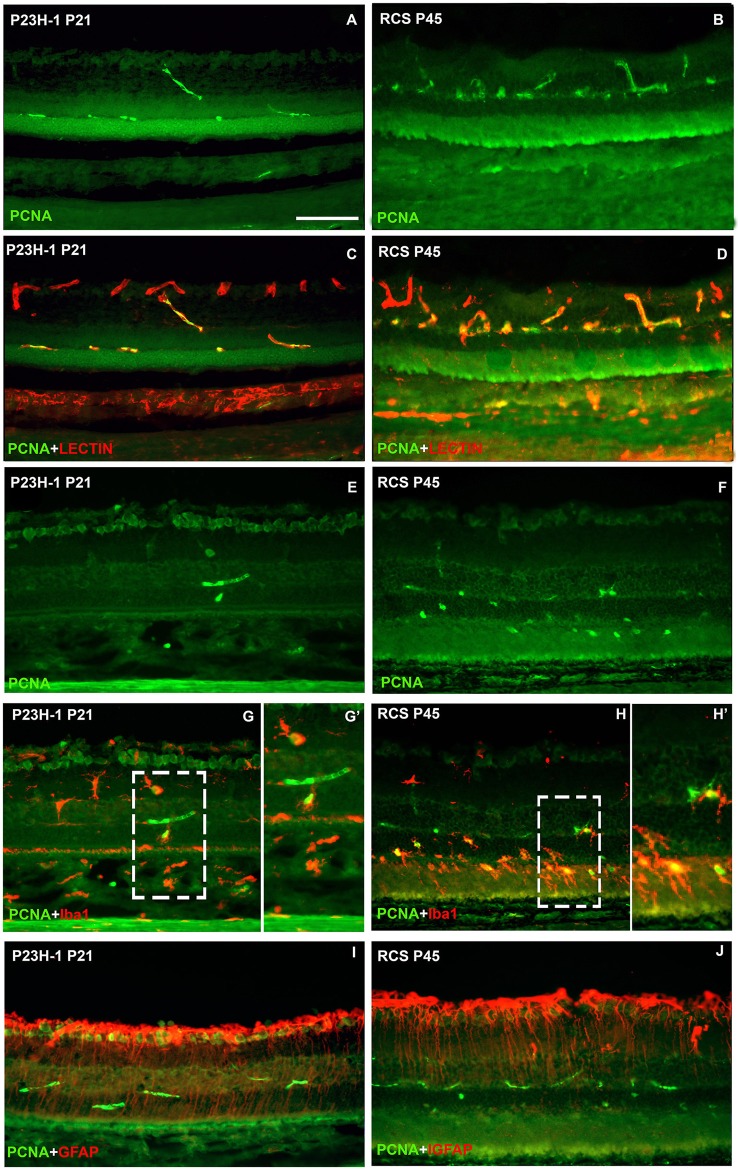
**Microglial cell increase is due to cellular division**. Photomicrographs from representative retinal cross sections of P21 P23H-1 rats (left column) and P45 RCS rats (right column) immunoreacted with antibodies against PCNA (cellular division), isolectin GS-IB4 (microglia and blood vessels) Iba1 (microglia) and GFAP (astrocytes and Müller cells). Note that the retina is thinner in P23H-1 rats due to disappearance of the photoreceptor outer segments. There was no expression of PCNA in control animals (not shown). In both dystrophic strains at these ages, PCNA was expressed in some blood vessels of the outer and inner retinal plexuses (green, **A–J**) that were double labeled with the lectin (red, **C,D**) and also in some microglial cells (green, **G,G',H,H'**) that were double labelled with anti-Iba-1 (red, **G,H,G',H'**) specific for microglial cells. PCNA could not be detected in Müller cells labeled with anti-GFAP antibody (red, **I,J**). **(G',H')** are insets from panels **(G,H)**, respectively. Scale bar = 100 μm.

To study whether the increase in microglial cell number is due to cellular division and/or migration, in retinal cross sections of P23H-1 and RCS rats immunodetection of PCNA, a marker of cellular proliferation was combined either with GS-IB4, a lectin that binds to microglia and blood vessels, Iba-1 or GFAP. In control animals no PCNA immunoreactivity was found. However, PCNA signal was consistently observed in the retinal blood vessels and also in microglial cells, but not in Müller cells, in both animal models, at two postnatal ages: P21 for P23H-1 rats and P45 for RCS rats (Figure 8). The numbers of microglial cells double labeled with the anti-PCNA antibody and with Iba-1 were very small in the P23H-1 rat (1–2 per section) but high (8–9 per section) in the RCS rat. These double labeled cells were situated in both models in the layers external to the inner nuclear layer (Figures 8G,H), although in the RCS rat many were in the photoreceptor outer segment layer and sometimes were in close proximity (Figure 8H). Therefore, our data suggest that the higher number of microglial cells found in dystrophic retinas and particularly in the RCS rat retina is due at least in part to cellular division of microglial cells.

## Discussion

**Figure 9:**
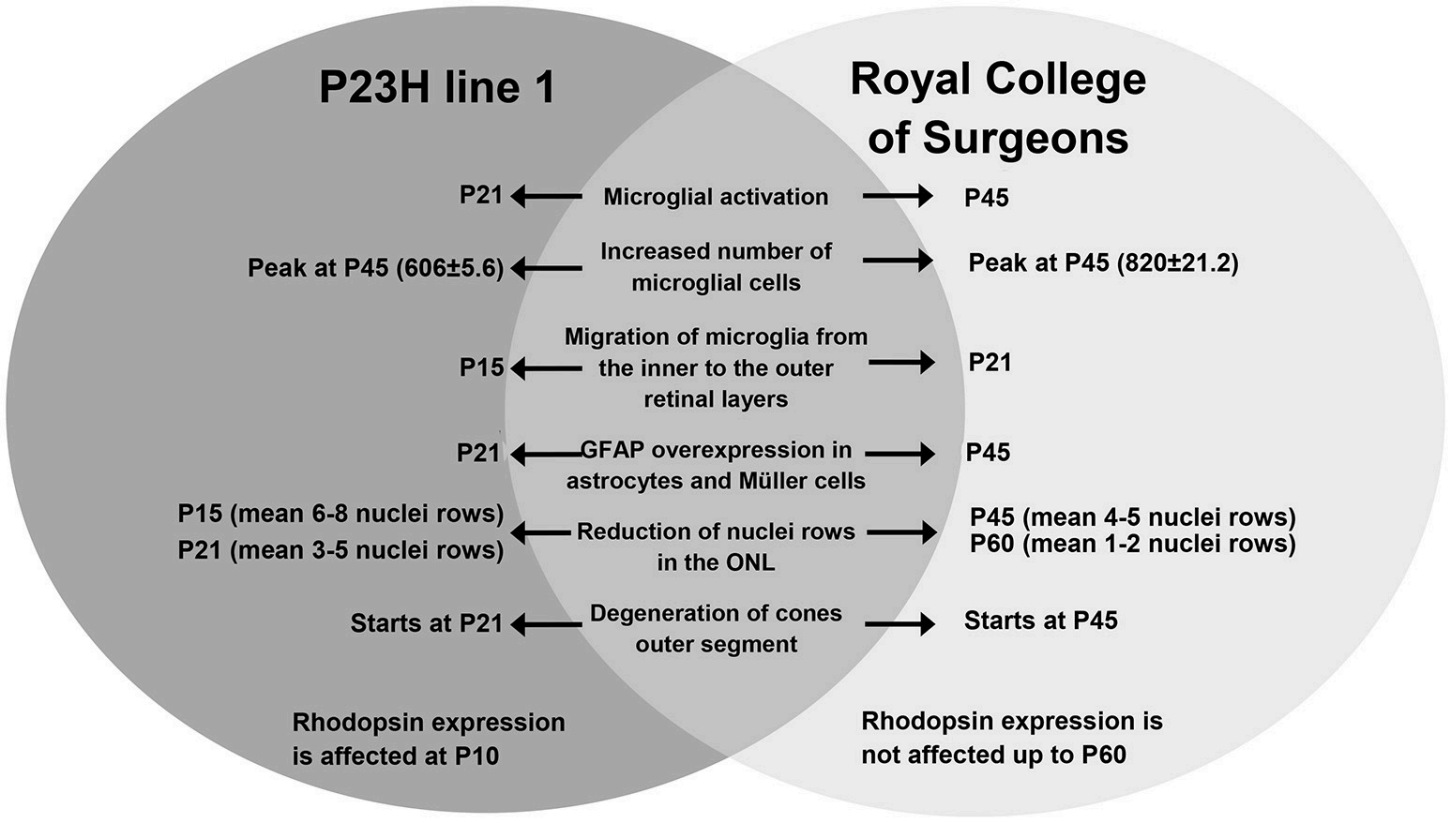
**Summary: comparison of the events taking place in the retina of P23H-1 and RCS rats**.

In this study, we have analyzed and compared the early events taking place in the retina in two animal models of RP caused by different etiologies, one affecting primarily rods, and the other affecting the pigmented epithelium and therefore both rods and cones. The similarities and differences between these two models are summarized in Figure 9, and they are discussed below.

### Control animals

In control rats, the density, shape and length of the OS of rods and cones was maintained at all ages, as previously reported (Ortín-Martínez et al., 2010; García-Ayuso et al., 2013). The thickness of the ONL varied between 8 and 12 nuclei and did not decrease with age, as reported in previous studies of this and other laboratories (García-Ayuso et al., 2010, 2011; Ortín-Martínez et al., 2015).

GFAP immunoreactivity in control animals was observed only in astrocytes of the innermost retinal layer as it has been reported for the healthy rat retina (Eisenfeld et al., 1984; Roque and Caldwell, 1990; Cuenca et al., 2014; Fernández-Sánchez et al., 2015; Vecino et al., 2016).

The microglial distribution in the different retinal layers in SD and PVG animals varied depending on age. At P21 in both strains microglial cells were located in five layers: NFL, GCL, IPL, INL, and OPL (Figures 3, 4) as previously reported for the adult rat retina (Terubayashi et al., 1984; Boya et al., 1987; Ashwell et al., 1989; Sobrado-Calvo et al., 2007). There were abundant microglial cells in the GCL and IPL, where their number remained constant, and there were few and decreasing with age in the INL and OPL. The dynamics of microglial cells in the INL and OPL at early postnatal ages could be due to the necessity of phagocytosis in these layers during the postnatal period of naturally occurring cell death. In fact, it has been documented that cell death is significant in the INL and ONL up to P15 and extends although at very low levels up to P40 (Braekevelt and Hollenberg, 1970; Beazley et al., 1987; Horsburgh and Sefton, 1987). Importantly, there were no microglial cells in the ONL and OS layers (Figures 4, 6, 7; Terubayashi et al., 1984; Boya et al., 1987; Ashwell et al., 1989; Sobrado-Calvo et al., 2007; Santos et al., 2008).

Microglial number decreases in rats (Ashwell et al., 1989) and mice (Santos et al., 2008) after the first post-natal week, and remains more or less constant at later ages (Noailles et al., 2016). In agreement with this, we found that microglial cells in control SD and PVG decreased significantly between P15 and P21 in SD rats and between P10 and P21 and between P21 and P33 in PVG rats, and thereafter their number did not change. Besides this different course, the other difference between both control strains was that at P10 and P21, PVG rats had significantly higher number of microglial cells than SD rats. The changes in the number and distribution of microglial cells with age shown here and in other studies indicate incomplete retinal development and migration of microglial cells (Denham, 1967; Braekevelt and Hollenberg, 1970; Beazley et al., 1987; Boya et al., 1987; Ashwell et al., 1989; Santos et al., 2008). Thus, we have interpreted our findings as evidence that the settlement of the adult microglial cell distribution is slower in PVG rats than in SD rats and we wonder whether there are differences in naturally occurring cell death between these two strains.

### Photoreceptor cell death during retinal degeneration

Photoreceptor cell death started at a different age and occurred at a very different pace in both models. In P23H-1 rats, photoreceptor cell death started at P15, while in RCS rats it did not commence until P33. Once started, the time course of cell death was different: in P23H-1 rats, cell death occurred from P15 to P21 and then stabilized (2–5 nuclei rows) and in RCS rats it was from P21 to P60 (<2 nuclei rows), the last age studied.

The decrease of nuclei rows in the ONL that we observed in dystrophic rats was similar to that observed in previous studies from our and other laboratories (Dowling and Sidman, 1962; Eisenfeld et al., 1984; Tso et al., 1994; García-Ayuso et al., 2010, 2011; Lu et al., 2013). However, in this study we show for the first time that microglial cell activation occurred in both models at around the same age at which photoreceptor death starts (P15 in P23H-1 animals and from P21 in RCS rats), and Müller cells gliosis (GFAP overexpression) occurs delayed after the initiation of photoreceptor cell death (see below).

### Affectation of the outer segments of photoreceptors during retinal degeneration

The different etiology of both models of retinal degeneration results in different morphologic changes of the outer segments of photoreceptors. We have used antibodies against rhodopsin and opsin to evaluate the outer segments of photoreceptors and, although in the P23H-1 rat, the rod outer segments were affected as soon as P10, in the RCS rat, the rod outer segments did not look affected at the last age studied in this work (P60). This is possibly due to the high density of rods and to the mechanism of cell death in RCS rats, where photoreceptor debris accumulate in the outer segment layer until they are removed long periods after degeneration has started (LaVail, 1981; Roque et al., 1996; Valter et al., 1998). Other reports using electron microscopy have documented alterations of the outer segments in RCS rats as early as P18 (Davidorf et al., 1991).

The difference in the degeneration observed in the outer rod segments between P23H-1 rats and RCS rats could be explained by the mechanisms of cell death and elimination of cell debris in the experimental models. Retinal degeneration in the P23H-1 rat is caused by a mutation in the rhodopsin gene (Steinberg et al., 1996; Machida et al., 2000), but the RPE is functional and therefore able to phagocytose the OS of dead photoreceptors. In the RCS rat the degeneration is caused by a defect in the MERKT gene that impedes retinal pigment epithelium phagocytosis and this results in the accumulation of photoreceptor OS “debris” (LaVail, 1981; Roque et al., 1996; Valter et al., 1998). The phagocytosis of the remaining OS is in the RCS rat assigned to microglial cells (Thanos, 1992; Thanos and Richter, 1993; Roque et al., 1996). This explains why the numbers of microglial cells are more abundant in the ONL and OS layers in RCS rats than in P23H-1 rats.

Although our qualitative findings were similar both for L- and S-opsin+ cones, cones were also differently affected in the two experimental models. In the P23H-1 rat, we observed cone morphologic changes at P21 but in RCS rats, similar changes were not observed until P45. Therefore, cones, similarly to rods, were also affected earlier in P23H-1 rats than in RCS rats. Cone survival in P23H rats depends on the mutation (Machida et al., 2000) and previous studies have documented cone degeneration from P21 in P23H-1 rats (Machida et al., 2000; Cuenca et al., 2004; Pinilla et al., 2005a; García-Ayuso et al., 2013), but also long term cone survival for P23H-3 rats (Chrysostomou et al., 2009; Lu et al., 2013; Fernández-Sánchez et al., 2015). In the RCS strain, previous electrophysiological and morphological studies have indicated that cone cell death may begin before P30 (Pinilla et al., 2005b; Rubin and Kraft, 2007; Huang et al., 2011).

### Microglial cell reaction in the degenerating retina

To label microglial cells in this study, we have immunodetected Iba-1, a protein present in microglial cells and macrophages (Ito et al., 1998). Using this antibody we could be labeling not only the retinal microglia, but also the macrophages that invade the retina and differentiate to microglial cells (Guillemin and Brew, 2004).

In accordance with the course of photoreceptor death, morphologic signs of microglial cell activation, increased densities and migration to outer layers of these cells occurred earlier in P23H-1 rats than in RCS rats. These data are in agreement to previous studies that have documented signs of microglial activation earlier in P23H-3 rats, a strain with a slower retinal degeneration than our P23H-1 rats, than in RCS and (Thanos and Richter, 1993; Roque et al., 1996; Liu et al., 2013; Noailles et al., 2014, 2016).

Previous studies in rats have not investigated the presence of microglial cell activation before the initiation of photoreceptor death, and to our knowledge only one study carried in rd10 mice has documented microglial activation before photoreceptor death (Roche et al., 2016). Therefore, this is the first study to investigate the relation between the initiation of photoreceptor death and microglial cell activation in rat models. Because we document that microglial cell activation occurs at approximately the same time as the initiation of photoreceptor cell death, we conclude that microglial cells increase in numbers, become activated and migrate to the outer layers as a response to photoreceptor death and thus, that microglial cell activation is a secondary event.

It has been postulated that the increased numbers of microglial cells in degenerating retinas may be due to division of resident microglial cells (Roque et al., 1996; Zeiss and Johnson, 2004; Liu et al., 2013) or invasion of macrophages from the blood or the ciliary epithelium (Garcia-Valenzuela and Sharma, 1999; Wohl et al., 2010). Cell division of resident microglial cells in inherited retinal degeneration has been suggested to occur in RCS rats (Roque et al., 1996), and documented in two models: P23H-3 rats (Noailles et al., 2016) and the rd-1 mice (Zeiss and Johnson, 2004). Because in this study we have observed proliferating microglial cells, it is possible that the increased numbers of microglial cells in both experimental models is due to microglial cell division, although it might be also in part due to macrophage migration from the blood or the subretinal space. In fact, breakdown of the blood-retinal barrier has been documented in these animal models (Zambarakji et al., 2006; Pinilla et al., 2016).

Two interesting findings of this study are that we have found microglial proliferation only at two ages: P21 for P23H-1 rats and P45 for RCS rats and that there are many more proliferating microglial cells in the RCS rat. It is possible that microglial cells proliferate at these ages because the rapid period of photoreceptor loss is almost finished and therefore more microglial cells are needed to phagozytose dead photoreceptors. Indeed, the proliferating microglial cells were located in the retinal layers external to the inner nuclear layer and in the RCS rat many of these cells were found in the photoreceptor outer segment layer. Other authors have also found proliferating microglial cells in the outer nuclear layer (Zeiss and Johnson, 2004) and in more internal retinal layers (Noailles et al., 2016) in mouse and rat retinal degenerative models, respectively.

### Macroglial response

In this study, we observed constant GFAP expression in astrocytes and a similar increased expression of GFAP in Müller cells with age in both animal models. However, this increased Müller cell immunoreactivity did not occur immediately after photoreceptor degeneration, but it was delayed for some time in both experimental models. This is not surprising because, in general, both in our study and in previous studies, it has been shown that microglial cells seem to be more sensitive than macroglial cells and react earlier to the pathologic events that take place during photoreceptor degeneration (Fernández-Sánchez et al., 2015; Roche et al., 2016). Müller cell activation and GFAP overexpression by these cells occurs practically in every retinal disease (Bringmann and Reichenbach, 2001; Bringmann et al., 2006), may reveal blood-retinal breakdown and pursue the restoration of normal retinal homeostasis. In animal models of inherited retinal degenerations, previous studies have shown GFAP overexpression both preceding (DiLoreto et al., 1995; Roche et al., 2016) or following (Eisenfeld et al., 1984; Ekström et al., 1988; Fernández-Sánchez et al., 2015) photoreceptor cell death in various inherited retinal degenerations and it has been postulated that it may be variable and depend on the etiology of the disease (Hippert et al., 2015). Our data does not support this because we observed similar increased expression in both models.

## Conclusions

We conclude that the different etiologies of both models of retinal degeneration result in different patterns of degeneration of the outer segments of photoreceptors and of glial activation and migration. Microglial cell activation occurs simultaneously with photoreceptor cell death, but GFAP overexpression by Müller cells is a delayed event. Also, microglial cell activation is accompanied by cell migration to the outer layers in both models, but the most external layers are invaded to a higher extent in RCS rats because the pigment epithelium is dysfunctional and therefore more microglial cells are needed for photoreceptor phagocytosis (Thanos, 1992; Thanos and Richter, 1993; Roque et al., 1996). Although microglial cells participate in photoreceptor cell death in virtually all models of retinal degeneration (Thanos, 1992; Thanos and Richter, 1993; Roque et al., 1996; Zhao et al., 2015), their role may not always be favorable as their inhibition has been documented to ameliorate some inherited retinal degenerations (Adamus et al., 2012; Iezzi et al., 2012; Peng et al., 2014; Zhao et al., 2015). Microglial cells activation and migration may thus have both beneficial and deleterious effects in retinal degenerations: they may have a positive effect though the phagocytosis of dead photoreceptors but at the same time a negative pro-inflammatory effect. It is thus possible that the retinal degenerative diseases in which microglial cells became more activated such as the RCS rat may have in the future a better chance for intervention by inhibiting microglial cells. This study provides information about the spatiotemporal activation of microglial cells in these animal models and may serve us as basis for future studies aimed to the neuroprotection of photoreceptors by inhibiting the activation of microglial cells.

## Ethics statement

Animal handling has been approved by the University of Murcia Ethics committee, and follows the guideline of ARVO for the use of animals in Vision Research, as disclosed in the manuscript.

## Author contributions

All authors have reviewed and approved the final version of this work. Conceptualized and designed the experiments: JD, DG, MA, MV, MPV. Performed the experiments: JD, DG. Data acquisition: JD, DG. Data analysis: JD, DG, MA, MV, MPV. Data interpretation, manuscript drafting: JD, DG, NC, IP, MA, MV, MPV. Contributed reagents/materials/analysis tools: MA, MV, MPV.

## Funding

Fundación Séneca, Agencia de Ciencia y Tecnología Región de Murcia (19881/GERM/15) and the Spanish Ministry of Economy and Competitiveness, Instituto de Salud Carlos III, Fondo Europeo de Desarrollo Regional “Una Manera de Hacer Europa” ISCIII-FEDER PI16/00380, PI16/00031, RD16/0008/0026, RD16/0008/0016, SAF2015-67643.

### Conflict of interest statement

The authors declare that the research was conducted in the absence of any commercial or financial relationships that could be construed as a potential conflict of interest.
